# Neutrophil Mobilization by Surface-Glycan Altered Th17-Skewing Bacteria Mitigates Periodontal Pathogen Persistence and Associated Alveolar Bone Loss

**DOI:** 10.1371/journal.pone.0108030

**Published:** 2014-09-16

**Authors:** Rajendra P. Settem, Kiyonobu Honma, Ashu Sharma

**Affiliations:** Department of Oral Biology, School of Dental Medicine, University at Buffalo, State University of New York, Buffalo, New York, United States of America; University of Florida, United States of America

## Abstract

Alveolar bone (tooth-supporting bone) erosion is a hallmark of periodontitis, an inflammatory disease that often leads to tooth loss. Periodontitis is caused by a select group of pathogens that form biofilms in subgingival crevices between the gums and teeth. It is well-recognized that the periodontal pathogen *Porphyromonas gingivalis* in these biofilms is responsible for modeling a microbial dysbiotic state, which then initiates an inflammatory response destructive to the periodontal tissues and bone. Eradication of this pathogen is thus critical for the treatment of periodontitis. Previous studies have shown that oral inoculation in mice with an attenuated strain of the periodontal pathogen *Tannerella forsythia* altered in O-glycan surface composition induces a Th17-linked mobilization of neutrophils to the gingival tissues. In this study, we sought to determine if immune priming with such a Th17-biasing strain would elicit a productive neutrophil response against *P. gingivalis*. Our data show that inoculation with a Th17-biasing *T. forsythia* strain is effective in blocking *P. gingivalis*-persistence and associated alveolar bone loss in mice. This work demonstrates the potential of O-glycan modified *Tannerella* strains or their O-glycan components for harnessing Th17-mediated immunity against periodontal and other mucosal pathogens.

## Introduction

The oral pathogen *Tannerella forsythia* and its cohabiting partner *Porphyromonas gingivalis* have been implicated in periodontitis, a polymicrobial disease that often leads to tooth loss in adults. These bacteria form biofilms in subgingival crevices (spaces between the gums and teeth) and instigate inflammatory responses destructive to the tooth supporting structures. In order to colonize and persist in the host environment, pathogens have constantly evolved strategies to modulate the innate as well as the adaptive immunity of the host. In this regard, the periodontal pathogen *T. forsythia* induce the development of Th2 responses, resulting in inflammatory alveolar bone loss [6]. This bacterium expresses a uniquely glycosylated surface envelope, known as the surface (S)-layer, which plays an immunomodulatory role in influencing the immunity [1]. The *T. forsythia* S-layer has recently been shown to be important in delaying the cytokine responses of the monocyte and macrophage cells *in vitro* [2]. More recently, we have shown that an O-glycan structural core linked to the bacterium's S-layer proteins is critical in shaping the cytokine responses of antigen-presenting cells and adaptive T cell immunity *in vivo* [3]. Furthermore, in a mouse model of periodontitis we demonstrated that a terminal pseudaminic acid and N-acetylmannosaminuronic containing tri-saccharide branch on an O-glycan core linked to the *Tannerella* surface proteins plays a role in dampening Th17 differentiation and mitigating neutrophil infiltration to the gingival tissue [3]. Likewise, *P. gingivalis*, has its own specific mechanisms proficient for evading and paralyzing the innate and adaptive immunity, and is considered a keystone pathogen whose colonization results in oral microbial dysbiosis [4]. The dysbiotic flora resulting from *P. gingivalis* colonization is thought to induce an exaggerated and dysfunctional immune response culminating in alveolar bone loss and other pathologies characteristic of periodontitis [4]. Targeting *P. gingivalis* is thus an attractive strategy to block disease pathogenesis. In this study we tested whether oral inoculation with an O-glycan altered *T. forsythia* strain ED1 [3], capable of inducing Th17-dependent neutrophil responses, would be efficacious in eradicating *P. gingivalis* in a mouse model. Our results demonstrate that oral inoculation with a Th17-biasing *T. forsythia* strain is able to confer protection against *P. gingivalis* colonization and associated alveolar bone loss.

## Materials and Methods

### Bacterial strains and culture conditions

*P. gingivalis* ATCC 33277, *T. forsythia* ATCC 43037 and *T. forsythia* ED1 inactivated in the gene encoding UDP-*N*-acetyl-mannosaminuronic dehydrogenase were grown in broth or agar plates containing 1.5% agar under anaerobic conditions as described previously [3], [5].

### Mice and bacterial inoculation

Specific-pathogen-free BALB/cJ mice (Jackson Laboratory, Bar Harbor, ME, USA), 5–6 weeks old (female) at the start of the experiment were maintained in the Laboratory Animal Facility of the University at Buffalo. Mice were maintained and handled in strict accordance with the recommendations in the Guide for the Care and Use of Laboratory Animals of the National Institutes of Health. The study protocol was approved by the Institutional Animal Care and Use Committee (IACUC) of the University at Buffalo (IACUC Approval # ORB05072Y). All efforts were made to minimize suffering and tissues were harvested after euthanizing animals with carbon dioxide inhalation. After 1-week quarantine period, mice were given kanamycin (1 mg/mL) in drinking water *ad libitum* for 5 days followed by a two-day antibiotic-free period to suppress the resident bacteria. Mice were divided into four groups (16 mice per group). The experimental protocol has been schematically summarized in Fig, 1. The control group received six doses of 200 µL of vehicle 1% carboxymethyl cellulose (CMC) without bacteria at 48 h intervals via oral gavage (group 1, sham-infected). Group 2 (control) received three doses of 10^8^ cfu *P. gingivalis* cells in 200 µL 1% CMC at 48 h intervals three days after priming with six doses of vehicle. Groups 3 and 4 were subjected to infection priming by oral gavage with six doses of 10^8^ cfu *T. forsythia* cells in 200 µL 1% CMC at 48 h intervals with either the live wild-type ATCC 43037 (groups 3) or trisaccharide O-glycan deficient *T. forsythia* ED1 strain (group 4). Three days after the last dose, *T. forsythia*-primed groups 3 and 4 were challenged with three doses of 10^8^ cfu *P. gingivalis* cells in 200 µL of 1% CMC at 48 h intervals as above.

**Figure 1:**
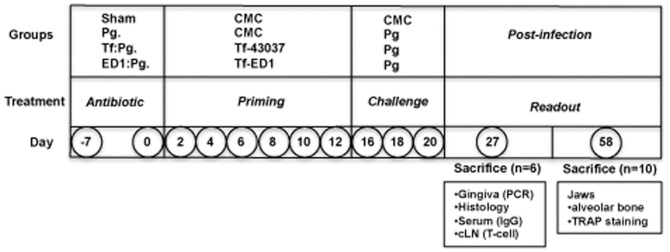
Schematic representation of infection and tissue harvesting schedules.

### Specific IgG response by ELISA

*T. forsythia* wild-type, ED1 and *P. gingivalis* strain specific IgG antibody responses were measured by ELISA as described previously [6]. Briefly, 96-well Immuno-Maxisorp plates (Nalgene Nunc International, Rochester, NY) coated with formalin-fixed bacteria (10^9^ cells/mL and 100 µL/well) were incubated with serial dilutions of mouse sera, followed by HRP-conjugated goat anti-mouse IgG (Bethyl Laboratories, TX). ELISA wells were color developed with TMB Micro well enzyme substrate (Kirkegaards and Perry, MD) and plates were read at 495 nm. Titers were defined as the log**^2^** of the highest dilution with a signal that was 0.1 optical density units above the background level.

### Assessment of alveolar bone loss

Horizontal bone loss around the maxillary molars was assessed by a morphometric method. After 6 weeks following the first infection mice were scarified, skulls were autoclaved, de-fleshed and immersed overnight in 3% hydrogen peroxide and then stained with 1% methylene blue as described previously [6]. The distances between the alveolar bone crest (ABC) and cement-enamel junction (CEJ) considered as alveolar bone loss were measured by two independent evaluators in blinded fashion at 7 buccal sites on each side with the help of a dissecting microscope attached to an imaging system with a software (Aquinto imaging system, a4i America, Brook-Anco, Rochester, NY).

### Intracellular staining and FACS analysis

The following Abs and isotype controls were purchased from e-Biosciences: PE-conjugated anti–IFN-γ and rat IgG2a; PE-conjugated anti–IL-5; PECy5-labeled anti-CD4; anti-CD80 (16-10A**1**) and anti-CD83 (Michel-17); rat IgG1, IgG2a, and IgG2b; PE-conjugated anti-CD11c (N418); hamster IgG; PE-conjugated anti–IL-17A (eBio17B7); and rat IgG2a. For intracellular staining, cell suspensions from cervical lymph nodes (cLNs) were stimulated with anti-CD3 and anti-CD28 Abs (eBioscience) for 48 h, followed by PMA (50 ng/ml), ionomycin (1 µg/ml), and brefeldin A (10 µg/ml) for 6 h. Cells were washed, incubated with FcR block (1 µg/ml; eBiosciences), and stained for CD4. Cells were fixed with 2% paraformaldehyde, permeabilized with 0.1% saponin (Sigma), and stained for IL-5, IL-17 and IFN-γ. Cells were analyzed on a FACS Calibur (BD Biosciences) with FCS Express software (DeNovo Software).

### Quantification of osteoclasts by TRAP staining

Mouse maxillary and mandibular bones (*n* = 4–6) were fixed in 10% phosphate-buffered formalin and decalcified in 10% EDTA. The samples were then embedded in paraffin, and sections at 4 µm were prepared and stained for tartrate-resistant acid phosphatase (TRAP; Sigma). TRAP-stained whole slides were digitally scanned immediately with a Scan Scope CS system (Aperio) to minimize color fading. The scanned slides were viewed with Image Scope viewing software (Aperio). The right maxillary and mandibular inter dental areas (average of 10 higher power fields per slide) of the crestal alveolar bone from the first molar to third molar were used to quantify osteoclasts.

### Quantification of CD45^+^ cells and infiltrated neutrophils

The right and left halves of the maxillary and mandibular bones with soft tissues were recovered and processed as follows. The left maxillary and mandibular tissues were used for DNA extraction (see below) and the right maxillary and mandibular tissues were used for histology after embedding in paraffin. Briefly, 4 µm sections were cut from the embedded tissues, deparafinized in xylene and hydrated in graded ethanol prior to antibody staining as follows. Specimens were first incubated at 90°C for 10 min with BD Retrievagen A (BD PharMingen) for antigen retrieval and then sequentially incubated in: (i) blocking solution containing 0.1% Triton X-100 in 0.1 M PBS (PBST) for 1 h at room temperature; (ii) monoclonal rat anti-mouse CD45 (BD PharMingen) antibody diluted 1∶30 in PBST or anti-neutrophil marker antibody (NIMP-R14, Santa Cruz Biotechnology, CA, USA) for 1 h at room temperature. The slides were incubated with a biotinylated secondary antibody (goat anti-rat IgG) followed by an avidin-biotin complex developed with 3, 3-diaminobenzidine (DAB) (Vector Labs, Burlingame, CA). The slides were counter stained with haematoxylin. After each step, slides were rinsed in PBST (3×10 min). Slides were scanned at an absolute magnification of 400× using the Aperio Scan Scope CS system (Aperio Technologies, Vista, CA). Slides (six slides per mouse) were viewed and analyzed using desktop personal computers employing the virtual image viewer software (Aperio). Antibody positive cells (stained brown) were enumerated manually in the inter-dental areas from the first to third molar at randomly selected locations in each slide (6 per slide). The areas associated with these locations were then obtained from the software to calculate the average cell densities per square millimeter.

### Assessment of bacterial colonization levels by real-time PCR

DNA was isolated from left maxillary and mandibular tissues (soft gingival and hard tissue) of each mouse a tissue DNA isolation kit (DNAeasy from Qiagen, Valencia, CA). *P. gingivalis* and *T. forsythia* levels in the tissues were determined by quantitative real-time PCR as described previously [3]. Briefly, *T. forsythia* or *P. gingivalis* specific 16S rDNA amplicons from tissue samples were amplified in a real-time PCR reaction with specific primer sets (*Tf*: 5′-GCGTATGTAACCTGCCCGCA-3′ and 5′-TGCTTCAGTTCAGTTATACCT-3′; *Pg*: 5′-CTTGACTTCAGTGGCGGCAG-3′ and 5′-AGGGAAGACGGTTTTCACCA-3′) and SYBR green master mix using MyiCycler (Bio-Rad laboratories). In addition, mouse glyceraldehyde-3-phosphate dehydrogenase (*gapdh*) gene amplified (primers 5-GCACAGTCAAGGCCGAGAAT-3 and 5-GCCTTCTCCATGGTGGTGAA-3) from the same sample was used to normalize bacterial loads in extracted tissues as per previously described method [3]; bacterial loads were expressed as the number of log_10_ bacterial genomes per 10^9^ copies of *gapdh*. Ct values were converted to weights of DNA by interpolation from DNA weights versus a Ct standard curve. For calculating the number of bacterial genomes from the quantities of DNA, the genome size of 3.4 Mb and 2.35 Mb for *T. forsythia* and *P. gingivalis* respectively (http://www.homd.org) were used.

### Data analysis

Data were analyzed with Graph Pad Prism software (Graph Pad, San Diego, CA). Comparisons between groups were made using a Student's *t* test (between two groups) or ANOVA (multiple group comparisons) as appropriate. Statistical significance was defined as *p*<0.05.

## Results

### Neutrophil mobilization to gingivae is enhanced and *P. gingivalis* persistence is moderately reduced in O-glycan-deficient ED1-primed mice

**Figure 2:**
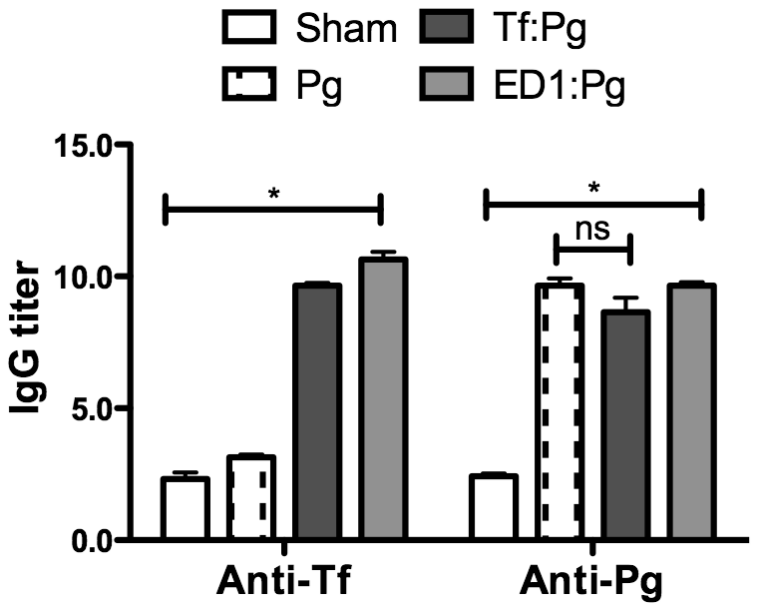
Antibody titers against *T. forsythia* and *P. gingivalis* after infection. Sera from mice 1-week after the last infection were analyzed by ELISA for *T. forsythia* and *P. gingivalis* specific IgG. Antibody levels are presented as log_2_ titers. Data are from one of two independent experiments with similar results (mean ± s.d, of 6 mice per group; *, *p*<0.05).

**Figure 3:**
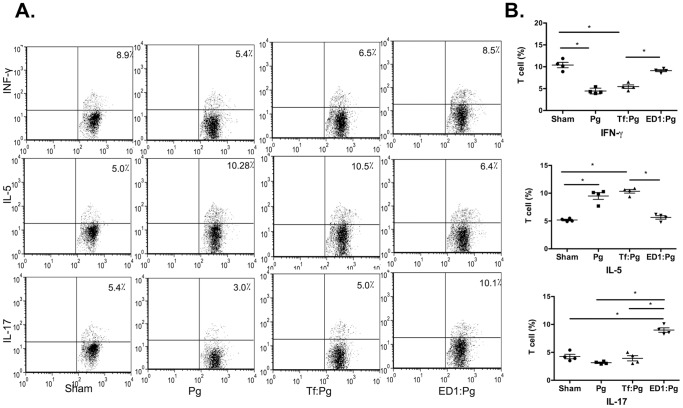
Flow cytometric analysis of cytokine production in CD4^+^ T cells from mice after infection. Draining lymph node cells from all groups were stimulated *in vitro* with anti-CD3 and anti-CD28 Abs for 48 h. Cells were then stimulated with PMA and ionomycin for 4–6 h prior to intracellular staining for IL-5, IFN-γ or IL-17 (A) Representative flow cytometry dot plots of CD4^+^ T cells from sham- and bacteria- infected mice intracellularly stained for IL-5, IFN-γ or IL-17. (B) Bar graphs showing percentages of specific cytokine positive T cells for each group. Data are from one of two independent experiments with similar results (mean ± s.d of 6 mice per group; *, *p*<0.05, n.s, not significant).

**Figure 4:**
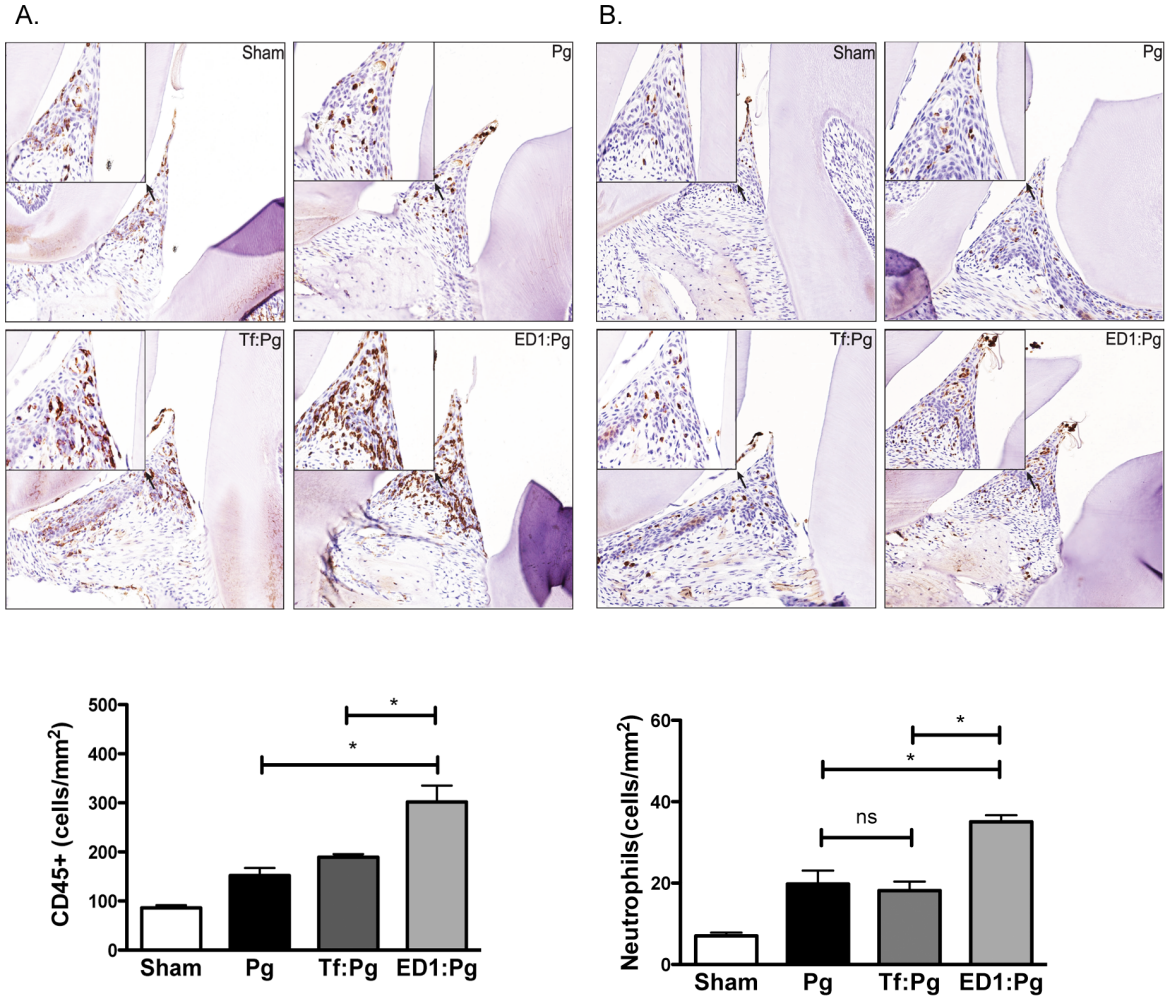
Lymphocyte/neutrophil infiltration in gingival tissues of mice primed with ED1 and infected with *P. gingivalis*. Immunohistochemical staining for total CD45 positive inflammatory cells (A) and neutrophils (B) in gingival tissue. All images are representative of 400× magnification. Slide images were viewed with Aperio Image Scope viewing software and the inter-dental areas from the first to third molar were used to quantify inflammatory cells. Bar graphs below each image show numbers of CD45 positive or neutrophil antibody positive cells (brown color). Data are from one of two independent experiments with similar results (mean ± s.d. of 6 mice per group; *, *p*<0.05).

**Figure 5:**
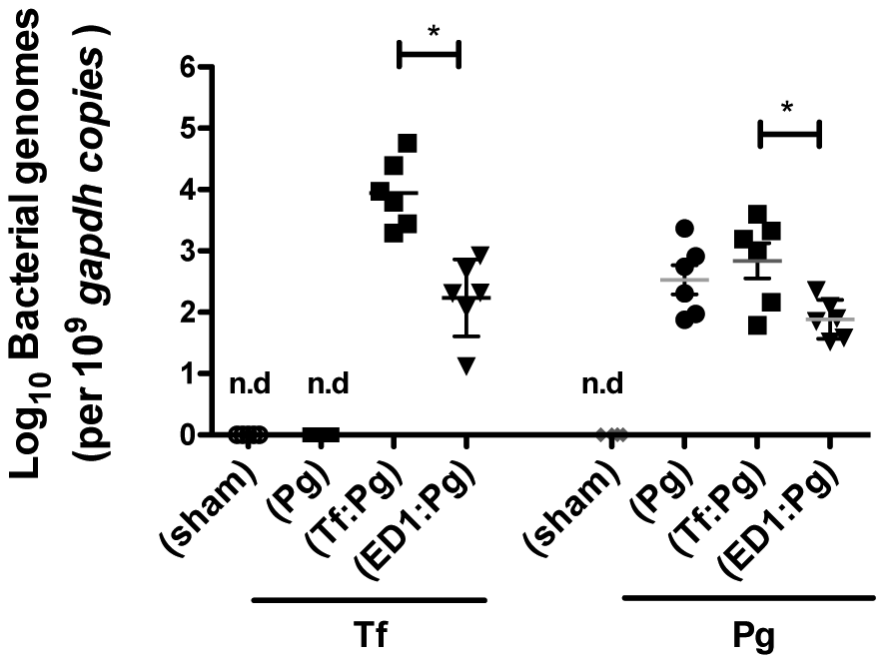
*P. gingivalis* persistence in the gingival tissues following priming with ED1. Bacterial loads in gingival tissues from mice were determined by quantitative PCR. Data are from one of two independent experiments with similar results (mean ± s.d. of 6 mice/group; *, *p*<0.05).

Previous studies from our laboratory showed that the O-glycans linked to the proteinaceous surface (S) - layer in the periodontal pathogen *T. forsythia* plays an immunomodulatory role in mice [3], [6]. Specifically, a *Tannerella* strain ED1 lacking the terminal trisaccharide motif comprising a modified pseudaminic acid and two *N*-acetylmannosaminuronic residues on a protein linked O-glycan core induces a Th17- response [3]. We tested whether a Th17-driven neutrophil response orchestrated by the ED1 strain would be productive in clearing *P. gingivalis*. Briefly, mice were inoculated with either the vehicle, wild-type *T. forsythia* or ED1 strains and then challenged with *P. gingivalis* as described (summarized in Fig. 1). Serum IgG titers against bacteria were also determined 1-week after the last infection dose to confirm that mice were productively infected. *T. forsythia* or *P. gingivalis*-specific IgG titers were significantly elevated in groups infected with the corresponding species over the sham-infected group (Fig. 2). Background antibody titers against *T. forsythia* or *P. gingivalis* in sham-infected groups were also observed, this likely reflects the presence of cross-reacting common antigens between these bacteria and the resident oral flora. Antibody titers against *T. forsythia* and *P. gingivalis* were also elevated in groups receiving both *T. forsythia* and *P. gingivalis* (Fig. 2). Th- cell response were evaluated by intracellular staining followed by flow cytometry on T cell populations from draining lymph nodes; single cell suspensions from cervical lymph nodes (cLNs) stimulated with anti-CD3, -CD28 antibodies and ionomycin were surface stained for cell surface marker CD4 and intracellularly for subset effector cytokines IL-5 (Th2), IFN-γ (Th1) and IL-17 (Th17). The results showed that the ED1-primed group (ED1: Pg) exhibited significantly higher levels of Th17 cells in cLNs compared to all other groups (Fig. 3). Mice infected with the wild-type *T. forsythia* and *P. gingivalis* (Tf:Pg) or *P. gingivalis* alone (Pg) showed an increase in the numbers of Th2 cells compared to the sham or ED1 infected mice (Fig. 3). To evaluate the inflammatory activity locally in the periodontal area, the left maxillae were collected, sectioned and stained with mouse specific CD45 and neutrophil (Gro^+^) antibodies as described previously [3]. The results showed that CD45^+^ inflammatory cells were elevated in all groups infected with bacteria compared to the control group (sham-infected). Moreover, the numbers of CD45^+^ cells were highest in the groups primed with ED1 (Fig. 4A). A similar trend was observed with neutrophils (Fig. 4B). ED1 primed and *P. gingivalis* challenged mice showed an increase in the numbers of Gro^+^ cells compared to all other groups (Fig. 4B). In addition, the level of neutrophil infiltration in each group paralleled the Th17 responses in the bacterial infected groups. Together these results indicated that mice primed with ED1 develop a sustained Th17-mediated neutrophil response which is not perturbed by *P. gingivalis* challenge (ED1:Pg). To evaluate if neutrophil infiltration due to ED1 was effective in clearing *P. gingivalis*, bacteria were quantified by quantitative PCR as described previously [3]. The results showed that the *P. gingivalis* colonization was significantly reduced in ED1- primed compared to the *T. forsythia* wild-type -primed mice (P<0.05). A trend toward reduced *P. gingivalis* colonization in the ED1-primed (ED1:Pg) group compared to the sham-primed (Pg) group was observed. However, this reduction in colonization did not reach the statistical significance (P = 0.067) (Fig. 5). With respect to *Tannerella*, the numbers of genomes recovered from the Tf:Pg group were approximately100-fold higher than the ED1:Pg group. This is likely due to the fact that ED1 is significantly more prone to capture and killing by antigen-presenting cells than the wild-type strain [3], No *P. gingivalis* or *T*, f*orsythia* were detected in sham-infected animals; Ct values higher than the cutoff set at 40 cycles and similar to template absent water control (Fig. 5).

### The O-glycan deficient ED1 strain protects mice from *P. gingivalis*-induced alveolar bone loss

**Figure 6:**
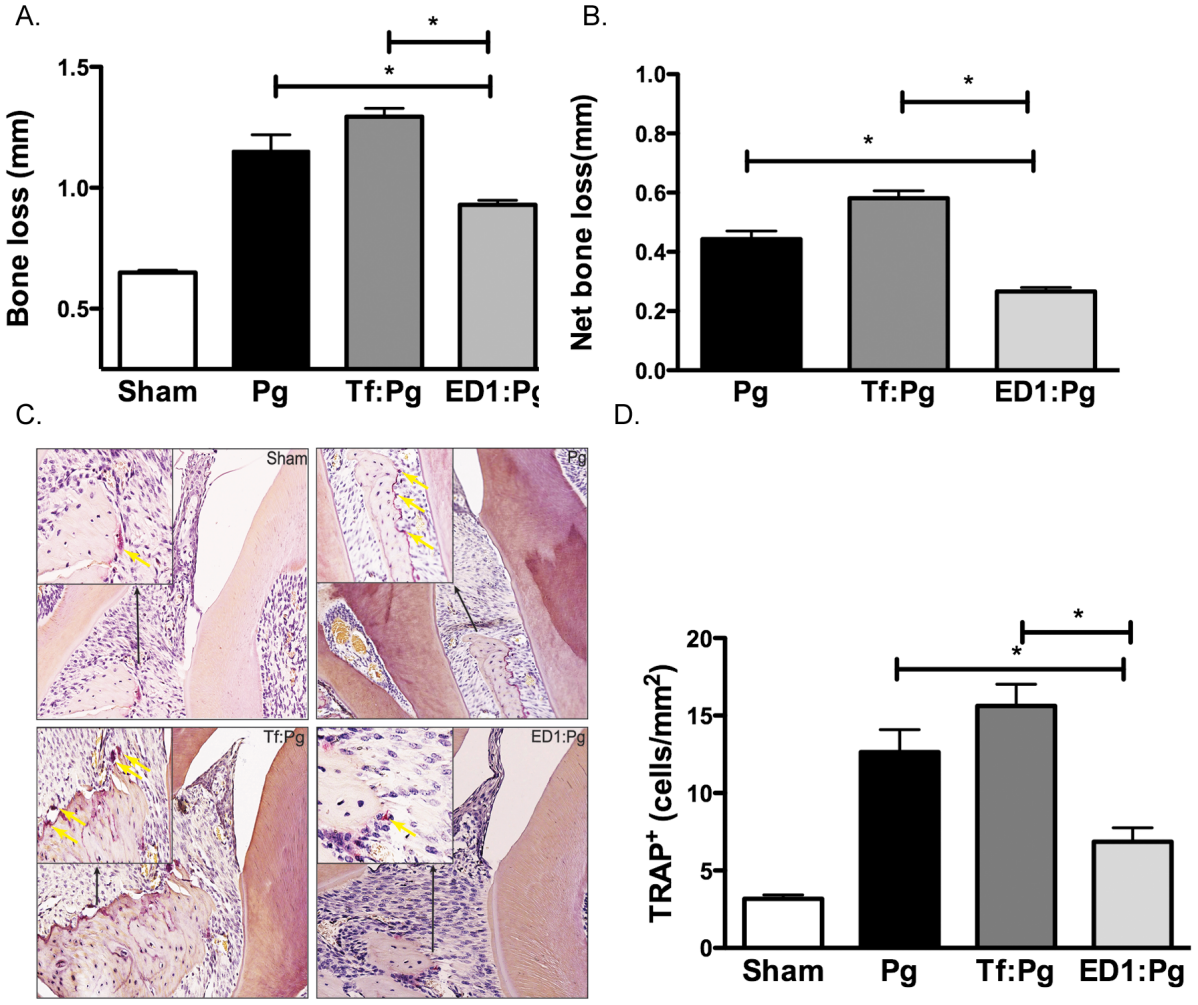
*P. gingivalis*-induced alveolar bone loss in mice primed with ED1. *A & B, alveolar bone levels*. A, Alveolar bone loss was assessed after 6 weeks by measuring the distance from the ABC to the CEJ at 14 maxillary buccal sites (7 sites on each side) per mouse and the total alveolar mean bone loss per group was plotted. B, Net bone loss induced by *P. gingivalis* in wild-type or ED1 primed groups was calculated as mean total ABC-CEJ distance of bacterially infected group minus mean total ABC-CEJ distance of the sham-infected group. *C & D*, osteoclastic activity measured as numbers of TRAP positive cells. C, Representative histological sections showing TRAP^+^ cells (indicated with yellow arrows) lining the bone crest in each group. (D) Average number of TRAP^+^ cells in 10 high power magnification fields/slide (4 mice/group). Data are from one of two independent experiments with similar results (mean ± s.d, of six mice in A & B or four mice in C & D; *, *p*<0.05).

To assess whether *P. gingivalis* clearance reciprocally reduces alveolar bone loss, mice jaws were harvested 6-weeks after the first *P. gingivalis* infection dose and the alveolar bone loss in each group was assessed as the average total distance between ABC-CEJ per mouse. The data showed that ED1 primed and *P. gingivalis* challenged mice (ED1:Pg) exhibited reduced alveolar bone loss as compared to the *T. forsythia* wild-type primed–*P. gingivalis* challenged (Tf:Pg) or the *P. gingivalis* alone challenged (Pg) mice (Fig. 6 A). The net bone loss calculated as the total mean bone loss in the sham infected group subtracted from the bacteria infected group was significantly less in the ED1:Pg group compared to the Pg alone or Tf:Pg groups. As an additional measure of alveolar bone loss, osteoclastic activity in the interdental region was evaluated for each group for tartrate-resistant alkaline phosphatase (TRAP) staining. The results showed that the TRAP activity in each group correlated with the alveolar bone loss of the corresponding group; ED1-primed mice exhibited lower osteoclastic activity compared to *T. forsythia* wild-type or *P. gingivalis* infected groups (Fig. 6 C&D).

## Discussion

It is well established that periodontal disease is a polymicrobial infection with the black-pigmented anaerobe *P. gingivalis* playing a key role alongside *T. forsythia* in periodontal disease and that *P. gingivalis* is adept in immune subversion strategies that allow the bacterium to persist in the host [7]. We sought to explore whether neutrophil mobilization to gingival tissue through oral inoculation with Th17-biasing strains of *T. forsythia* could impair *P. gingivalis* persistence in the oral cavity. *P. gingivalis* is a keystone pathogen whose presence results in the development of a dysbiotic sub-gingival microbial community [4], triggering a dysregulated immune response culminating in inflammatory alveolar bone destruction, a hallmark of periodontitis. *P. gingivalis* utilizes a plethora of strategies to weaken the host innate and adaptive immunity that allow the bacterium to increase its colonization potential and existence in the oral cavity [7]. We reasoned that approaches by which neutrophil recruitment to the gingival tissues could be enhanced would be beneficial in clearing *P. gingivalis*, and thus in mitigating inflammatory alveolar bone loss. Our results demonstrated that by priming with Th17-biasing *T. forsythia* ED1 strain a productive neutrophil response able to block *P. gingivalis* colonization and associated alveolar bone loss was achieved in a mouse model. As shown, increased Th17-linked neutrophil infiltration into the gingival epithelium was observed in mice primed with the O-glycan altered *T. forsythia* ED1 strain, which also led to *P. gingivalis* clearance from the gingival tissues and reduced alveolar bone loss.

Here we utilized an attenuated strain of *T. forsythia* ED1 to induce a Th17 dependent neutrophil response able to clear *P. gingivalis*. ED1 lacks a trisaccharide motif consisting of a pseudaminic acid with two mannosaminuronic residues at the terminal end of an O-glycan core linked to the bacterial surface-layer proteins via serine threonine residues [8]. While the exact mechanism by which the O-glycan trisaccharide motif in *T. forsythia* modulates T cell immunity is not yet known, we envision that these O-glycans differentially regulate C-type lectin receptor(s) signaling to orchestrate the expression of T cell differentiation cytokines in dendritic cells and macrophages [1]. In general, a clear mechanistic understanding of how bacterial polysaccharides regulate T cell polarization is currently lacking because polysaccharide antigens are thought to be T cell independent antigens. However, the orchestration of T cell immunity via microbe associated glycans has been observed in other settings as well. For example surface capsular polysaccharide modified with methyl phosphoramidate in *Campylobacter jejuni* has been shown to modulate Th17 immunity against the pathogen and its persistence in the host [9]. The polysaccharide antigen (PSA) expressed by the ubiquitous human gut commensal and opportunistic pathogen *Bacteroides fragilis* down regulates Th17 differentiation and promotes IL-10-producing FoxP3^(+)^ Treg cells in the gut mucosa [10]–[12]. Similarly, polysaccharides with immunomodulatory roles have also been identified in bacteria such as *Streptococcus pneumoniae* and *Staphylococcus aureus* type-5 and 8 [13], [14].

In the gingival sulcus, neutrophils and Th17 responses play protective roles by clearing *P. gingivalis*. Neutrophils are sentinel cells of the innate immune system that keep the gingival surface free of pathogenic bacteria by forming a protective ‘wall’ [15], [16]. At the same time, a heightened neutrophil infiltration could be detrimental to the host due to the toxic effects of neutrophil components [16], [17]. Thus, the regulation of neutrophil function is critical in determining the severity and extent of periodontal inflammation. Likewise, IL-17 producing Th17 cells have been associated with both immune pathology and protection against infectious diseases. Th17 cells play an essential role in protective immunity against pathogens by coordinating early neutrophil recruitment into local sites of infection [18]. IL-17, in addition to orchestrating the chemokine cascades required for neutrophil mobilization, may also augment the killing activity of neutrophils even in the absence of pathogen opsonization with antibody or complement [19]. IL-17 driven neutrophil mobilization has been demonstrated to be critical in protection against *P. gingivalis* induced alveolar bone loss in a mouse model [20]. On the other hand, IL-17 produced byTh17 cells themselves can drive periodontal bone loss in chronic *P. gingivalis* infections [21]. In chronic settings neutrophils themselves can also become a source of IL-17, leading to IL-17-dependent inflammatory bone loss [22]. However, the complex role of IL-17 (Th17) in periodontitis remains to be determined [23]. A number of strategies that focus on exploiting host responses for the treatment of periodontitis are currently being explored. These include, but are not limited to, vaccine strategies to block *P. gingivalis* colonization [24] and targeted therapeutics modulating inflammatory cascades regulated by complement [25], glycogen synthase kinase 3β signaling [26] and T regulatory cells [27]. Bacterial S-layer proteins self-assemble *in vitro* to form nanoaggregates [28], which have been proposed as vehicles for vaccine antigen or therapeutic delivery. We envision that S-layer nanoaggregates from the ED1 strain could be directly delivered to gingival mucosa for the induction of local Th17 immunity for the eradication of *P. gingivalis*. We caution that all of the strategies described aimed at modulating host inflammatory responses require a careful analysis to weigh their associated advantages and risks. For instance anti-inflammatory therapies might lead to the colonization of pathogens such as *Candida* sp. whose colonization and virulence are kept in check via Th17 responses.

## References

[1] Settem RP, Honma K, Stafford GP, Sharma A (2013) Protein-linked glycans in periodontal bacteria: prevalence and role at the immune interface. Front Microbiol 4 PMID: 24146665 (open access) 10.3389/fmicb.2013.00310 PMC3797959 24146665

[2] Sekot G, Posch G, Messner P, Matejka M, Rausch-Fan X, et al (2011) Potential of the Tannerella forsythia S-layer to delay the immune response. J Dent Res 90: 109–114. 20929722 10.1177/0022034510384622 PMC4382719

[3] Settem RP, Honma K, Nakajima T, Phansopa C, Roy S, et al (2012) A bacterial glycan core linked to surface (S)-layer proteins modulates host immunity through Th17 suppression. Mucosal Immunol 6: 415–426. 22968422 10.1038/mi.2012.85 PMC4049606

[4] Hajishengallis G, Darveau RP, Curtis MA (2012) The keystone-pathogen hypothesis. Nat Rev Microbiol 10.1038/nrmicro2873 PMC3498498 22941505

[5] Honma K, Mishima E, Inagaki S, Sharma A (2009) The OxyR homologue in *Tannerella forsythia* regulates expression of oxidative stress responses and biofilm formation. Microbiology 155: 1912–1922. 19389765 10.1099/mic.0.027920-0 PMC2782426

[6] Myneni SR, Settem RP, Connell TD, Keegan AD, Gaffen SL, et al (2011) TLR2 signaling and Th2 responses drive *Tannerella forsythia*-induced periodontal bone loss. Journal of Immunology 187: 501–509. 10.4049/jimmunol.1100683 PMC3119786 21632710

[7] Hajishengallis G, Lamont RJ (2014) Breaking bad: Manipulation of the host response by *Porphyromonas gingivalis* . Eur J Immunol 44: 328–338. 24338806 10.1002/eji.201344202 PMC3925422

[8] Posch G, Pabst M, Brecker L, Altmann F, Messner P, et al (2011) Characterization and scope of S-layer protein O-glycosylation in *Tannerella forsythia* . J Biol Chem 286: 38714–38724. 21911490 10.1074/jbc.M111.284893 PMC3207478

[9] Maue AC, Mohawk KL, Giles DK, Poly F, Ewing CP, et al (2012) The polysaccharide capsule of Campylobacter jejuni 81–176 modulates the host immune response. Infect Immun 10.1128/IAI.01008-12 PMC3584872 23250948

[10] Surana NK, Kasper DL (2012) The yin yang of bacterial polysaccharides: lessons learned from B. fragilis PSA. Immunol Rev 245: 13–26. 22168411 10.1111/j.1600-065X.2011.01075.x PMC3243960

[11] Ochoa-Reparaz J, Mielcarz DW, Wang Y, Begum-Haque S, Dasgupta S, et al (2010) A polysaccharide from the human commensal Bacteroides fragilis protects against CNS demyelinating disease. Mucosal Immunol 3: 487–495. 20531465 10.1038/mi.2010.29

[12] Mazmanian SK, Round JL, Kasper DL (2008) A microbial symbiosis factor prevents intestinal inflammatory disease. Nature 453: 620–625. 18509436 10.1038/nature07008

[13] Gonzalez-Fernandez A, Faro J, Fernandez C (2008) Immune responses to polysaccharides: lessons from humans and mice. Vaccine 26: 292–300. 18160186 10.1016/j.vaccine.2007.11.042

[14] Tzianabos AO, Finberg RW, Wang Y, Chan M, Onderdonk AB, et al (2000) T cells activated by zwitterionic molecules prevent abscesses induced by pathogenic bacteria. J Biol Chem 275: 6733–6740. 10702228 10.1074/jbc.275.10.6733

[15] Darveau RP (2010) Periodontitis: a polymicrobial disruption of host homeostasis. Nat Rev Microbiol 8: 481–490. 20514045 10.1038/nrmicro2337

[16] Scott DA, Krauss J (2012) Neutrophils in periodontal inflammation. Front Oral Biol 15: 56–83. 22142957 10.1159/000329672 PMC3335266

[17] Nussbaum G, Shapira L (2011) How has neutrophil research improved our understanding of periodontal pathogenesis? Journal of Clinical Periodontology 38 Suppl 11: 49–59. 21323704 10.1111/j.1600-051X.2010.01678.x

[18] Curtis MM, Way SS (2009) Interleukin-17 in host defence against bacterial, mycobacterial and fungal pathogens. Immunology 126: 177–185. 19125888 10.1111/j.1365-2567.2008.03017.x PMC2632692

[19] Lu YJ, Gross J, Bogaert D, Finn A, Bagrade L, et al (2008) Interleukin-17A mediates acquired immunity to pneumococcal colonization. PLoS Pathog 4: e1000159. 18802458 10.1371/journal.ppat.1000159 PMC2528945

[20] Yu JJ, Ruddy MJ, Wong GC, Sfintescu C, Baker PJ, et al (2007) An essential role for IL-17 in preventing pathogen-initiated bone destruction: recruitment of neutrophils to inflamed bone requires IL-17 receptor-dependent signals. Blood 109: 3794–3802. 17202320 10.1182/blood-2005-09-010116 PMC1874584

[21] Moutsopoulos NM, Kling HM, Angelov N, Jin W, Palmer RJ, et al (2012) *Porphyromonas gingivalis* promotes Th17 inducing pathways in chronic periodontitis. J Autoimmun 39: 294–303. 22560973 10.1016/j.jaut.2012.03.003 PMC3416947

[22] Hajishengallis E, Hajishengallis G (2013) Neutrophil Homeostasis and Periodontal Health in Children and Adults. J Dent Res 10.1177/0022034513507956 PMC3929973 24097856

[23] Cheng WC, Hughes FJ, Taams LS (2014) The presence, function and regulation of IL-17 and Th17 cells in periodontitis. J Clin Periodontol 10.1111/jcpe.12238 24735470

[24] Choi JI, Seymour GJ (2010) Vaccines against periodontitis: a forward-looking review. J Periodontal Implant Sci 40: 153–163. 20827324 10.5051/jpis.2010.40.4.153 PMC2931303

[25] Hajishengallis G, Lambris JD (2013) Complement-targeted therapeutics in periodontitis. Adv Exp Med Biol 734a: 197–206. 10.1007/978-1-4614-4118-2_13 PMC3466049 23402028

[26] Adamowicz K, Wang H, Jotwani R, Zeller I, Potempa J, et al (2012) Inhibition of GSK3 abolishes bacterial-induced periodontal bone loss in mice. Mol Med 18: 1190–1196. 22847803 10.2119/molmed.2012.00180 PMC3510296

[27] Glowacki AJ, Yoshizawa S, Jhunjhunwala S, Vieira AE, Garlet GP, et al (2013) Prevention of inflammation-mediated bone loss in murine and canine periodontal disease via recruitment of regulatory lymphocytes. Proc Natl Acad Sci U S A 110: 18525–18530. 24167272 10.1073/pnas.1302829110 PMC3831997

[28] Ilk N, Egelseer EM, Sleytr UB (2011) S-layer fusion proteins–construction principles and applications. Curr Opin Biotechnol 22: 824–831. 21696943 10.1016/j.copbio.2011.05.510 PMC3271365

